# Machine intelligence for nerve conduit design and production

**DOI:** 10.1186/s13036-020-00245-2

**Published:** 2020-09-09

**Authors:** Caleb E. Stewart, Chin Fung Kelvin Kan, Brody R. Stewart, Henry W. Sanicola, Jangwook P. Jung, Olawale A. R. Sulaiman, Dadong Wang

**Affiliations:** 1 Current Affiliation: Department of Neurosurgery, Louisiana State University Health Sciences Center, Shreveport Louisiana, USA; 2 Current Affiliation: Department of General Surgery, Brigham and Women’s Hospital, Boston, MA 02115 USA; 3 Current Affiliation: Department of Surgery, Mayo Clinic College of Medicine, Rochester, MN 55905 USA; 4 Department of Biological Engineering, Louisiana State University, Baton Rouge, LA 70803 USA; 5 Ochsner Neural Injury & Regeneration Laboratory, Ochsner Clinic Foundation, New Orleans, LA 70121 USA; 6 Department of Neurosurgery, Ochsner Clinic Foundation, New Orleans, 70121 USA; 7 Quantitative Imaging Research Team, Data 61, Commonwealth Scientific and Industrial Research Organization, Marsfield, NSW 2122 Australia

**Keywords:** Bioprinting, Data science, Tissue engineering, Computer vision, Nerve regeneration, Machine learning, Artificial intelligence

## Abstract

Nerve guidance conduits (NGCs) have emerged from recent advances within tissue engineering as a promising alternative to autografts for peripheral nerve repair. NGCs are tubular structures with engineered biomaterials, which guide axonal regeneration from the injured proximal nerve to the distal stump. NGC design can synergistically combine multiple properties to enhance proliferation of stem and neuronal cells, improve nerve migration, attenuate inflammation and reduce scar tissue formation. The aim of most laboratories fabricating NGCs is the development of an automated process that incorporates patient-specific features and complex tissue blueprints (e.g. neurovascular conduit) that serve as the basis for more complicated muscular and skin grafts. One of the major limitations for tissue engineering is lack of guidance for generating tissue blueprints and the absence of streamlined manufacturing processes. With the rapid expansion of machine intelligence, high dimensional image analysis, and computational scaffold design, optimized tissue templates for 3D bioprinting (3DBP) are feasible. In this review, we examine the translational challenges to peripheral nerve regeneration and where machine intelligence can innovate bottlenecks in neural tissue engineering.

## Background—critical challenges in (re)innervation

Peripheral nerve injuries (PNI) are a common source of disability that originate from traumatic, nontraumatic, and iatrogenic causes [1–3]. Advancements made in tissue engineering have led to the emergence of nerve guidance conduits (NGC) that offer a promising replacement for autografts [4]. Nerve guides are tubular biostructures designed to house growth factors and neural progenitor stem cells in a microenvironment conducive for nerve regeneration. Many challenges exist in clinical research to produce a conduit that meets or exceeds the performance of autografts for treatment of short and long gap nerve injuries. From a clinical research standpoint, Sun et al. have documented a recent rise in randomized control trials (RCTs) for peripheral nerve repair but suboptimal quality of systematic reviews on the subject [5]. They found the number of annual systematic reviews increased from 2004 to 2015 but median scores rated fair in quality throughout this period [5]. Establishing NGC superiority over traditional treatments will be a major challenge considering evidence-based medicine (EBM) faces similar problems determining the effectiveness of standard peripheral nerve repair [5, 6]. In particular, PNI categories cover a large domain that includes nerve types (sensory, motor, both), mechanisms (stretch, crush, percussion, laceration), anatomical regions (plexuses, nerve root, extremities), and anatomical variants. Tissue engineers need to consider these standards among others when designing nerve conduits and scaffolds. This makes a transdisciplinary approach attractive since experts from the fields of engineering, physics, computer science, and medicine can blend their faculties into a comprehensive biomanufacturing process and product.

Nerve conduits provide a customizable solution for both repair options by tailoring conduit features to patient-specific injuries to enhance the repair of acute or chronic injuries. Narayan et al. performed a systematic review and meta-analysis of three randomized control trials (RCTs) comparing conduits with conventional nerve repair [7]. Three RCTs showed conduits were significantly more effective compared to standard end-to-end suture repair for short gap (< 10 mm) sensory nerve injuries [7]. More studies are required to assess the effectiveness of conduits for motor and mixed nerves including cranial nerves and complex nerve plexuses.

From a tissue engineering perspective, many novel features have been utilized for peripheral nerve regeneration [8], specifically, neurotrophic factors and anisotropic gradients, electrical stimulation [9], pluripotent stem cell derived progenitor cells [10, 11], 3DBP [12], immuobioengineering [13], nerve differentiation strategies, simultaneous vascularization, design customization [14], and gene therapy [15–17]. Here we briefly reviewed to identify points of intersection between tissue engineering and machine intelligence with a particular concentration on peripheral nerve regeneration. We utilize these examples along with current engineering challenges encountered in peripheral nerve regeneration to postulate where machine intelligence can complement biofabrication in product performance, additive manufacturing (AM) processes, and medical regulatory compliance.

### Classification of nerve injuries

**Fig. 1:**
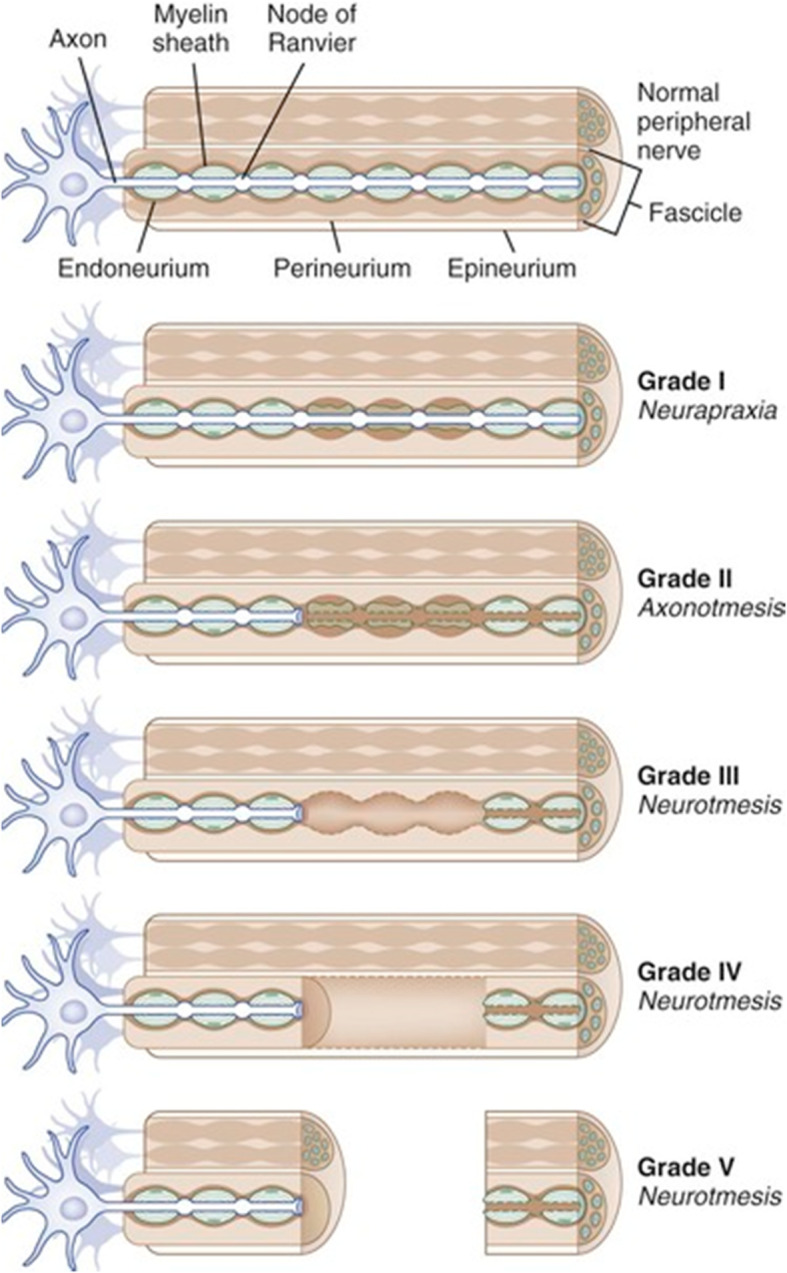
Sunderland Classification of Nerve Injuries [18]

In 1951, the Sunderland classification system became (Fig. 1) the preferred PNI grading system, since it makes better clinical prognostications and directs appropriate therapy [19–21]. Sunderland identified five injury grades where Grade I is the least severe and Grade 5 is the most severe. Grade I is neuropraxia caused by focal demyelination and presents with short term paralysis. Neuropraxia is a reversible injury that does not require intervention. Whereas, Grade II corresponds to axonotmesis denoting axonal destruction with intact epineurium [22, 23]. Axonotmesis is an irreversible injury to the axon usually caused by crush, stretch or percussive events. Grade III PNI results in the loss of endoneurium but intact perineurium leading to misguided axonal regeneration and making spontaneous recovery less probable [24, 25]. This injury is referred to as the least severe form of neurotmesis. Grade III injuries recover partially and do not require surgical repair. Grades IV and V represent PNIs requiring surgical intervention to restore function. Grade IV are the result of damage to the endoneurium and perineurium. While, Grade V is loss of all three layers ensheathing the nerve [22, 25]..

(Reproduced with permission from reference 19. Copyright Elsevier [18])

### Current strategies of NGC Design in Tissue Engineering

Here we categorize current strategies of NGC design with integrating: 1) biological modulation 2) engineering approaches and 3) surgical intervention.

### Biological modulation

- Gene delivery

Gene therapy has developed novel therapeutics to treat peripheral nerve insults [26]. Gene therapeutic strategies extend to but are not limited to eliminating toxic proteins at injured sites, activating regenerative phenotypes in chronic nerve injuries, increasing expression of therapeutic signals in cellular components in nerve regeneration, increasing sensitivity to cell to cell communication, and programming stem cells to differentiate in a specific manner [26]. Novel studies have improved transduction efficiency and genetic transfer using both non-viral and adenovirus methods [27–29]. Adeno-associated viral (AAV) vectors have become a popular method for gene delivery in peripheral nerves because of their low risk for immunogenicity, mutagenesis, and higher titers [30–32]. Different viral serotypes display unique transduction profiles and perform better in specific neurons [33, 34]. For example, AAV5 is the preferred serotype for treating sensory neurons in rat models [34]. AAV vectors have been effective tools for identifying the effects of particular genes on regeneration and survival of motor and sensory neurons [35–38].

Gene therapy can target Schwann cells whose regenerative properties fade after chronic states of denervation [29]. Gene therapy studies have typically used lentiviral vectors to enhance regenerative phenotypes of Schwann cells seeded in nerve guides [39–43]. Gene therapy targets neurotrophic factors (NTF) genes to increase their expression, stimulate axon regeneration, and promote directional growth towards targets [39, 44, 45]. One drawback to this method is overexpression of NTFs and trapping of budding axons [43, 46]. These findings require future research to optimize the concentration and temporal gene expression to maximize therapeutic potential. Further research is required to develop vector modalities, improve vector safety, and identify gene targets.

b. Growth factor stimulation

Growth factor (GF) for stimulation of peripheral nerve regeneration is not a new concept. NTFs are placed in the lumen of nerve guides to promote regeneration. Effective factors for nerve regeneration include nerve growth factor (NGF), neurotrophin-4/5, neurotrophin-3 (NT-3), glial cell derived neurotrophic factor (GDNF), ciliary neurotrophic factor (CNTF), fibroblast growth factor (FGF), interleukin-6 (IL-6), and brain derived neurotrophic factor (BDNF) [47–51]. NTFs use pathfinding gradients to stimulate or inhibit axonal outgrowth, [52, 53] direct axonal elongation [54], promote stem cell differentiation [55] and recruitment [56], and program Schwann cells [57]. Several design strategies have been employed by directly conjugating NTFs to conduit walls [58] or stimulating neural cells to secrete NTFs within the lumen [59, 60]. Another GF strategy arranges NTFs, extracellular matrix (ECM) proteins, and stem cells in a longitudinal gradient to orient regenerating axons towards the distal stump. NGCs are beginning to incorporate anisotropic gradients into their designs, which mimic physiological conditions for nerve injury repairs [49, 52, 61]. Several problems arise when working with NTFs including: determining optimal NTF dosing, NTF mixtures, biological gradients, and release kinetics confining their use to experimental research [62–64]. The uncertainty surrounding GF variables, GF limited stability (short half-life), and regenerative interference at high dosages limit translation into surgical products [65]. Finding strategies to address these challenges would make 3D bio-printed products more effective, feasible, and predictable for clinical use [66].

c. Autologous and allogeneic stem cells

Stem cells mimic physiological repair responses known as Büngner bands when arranged in NGC microarchitecture [67], thus incorporating stem cells into NGC design is a promising strategy to promote nerve regeneration. Autologous stem cells are difficult to harvest and proliferate in a time-sensitive manner making non-autologous sources more attractive options for nerve lacerations [68, 69]. However, patient-derived stem cells have the advantages in that they do not provoke an immune response, which avoids expensive phase I and II toxicity and biocompatibility tests and lowers the regulatory burden for advanced therapy medicinal products (Regulation (EC) 1394/2007). Autologous cells would work well for delayed repairs in blunt trauma cases or with poor recovery following a period of observation. The time waiting for surgery would allow for adequate cell harvest and maturation time for eventual conduit seeding. Popular autologous cells include skin-derived neural precursors, bone marrow-derived stem cells (BMSCs), nerve stem cells (NSCs), adipose-derived stem cells (ADSCs), and pluripotent stem cells (PSCs) [70, 71]. Stem cells can be selected for specific phenotypes having a predilection for motor or sensory differentiation [72]. Thus, seeding lumens with specific cell phenotypes in conduit lumens can generate motor and sensory tracts in mixed conduits. Each stem cell group has its advantages and drawbacks. BMSCs have been proven as effective as autografts in animal models for nerve regeneration [73]. However, harvesting these cells is uncomfortable with poor differentiation and proliferation [74]. NSCs are even more complex to harvest and have an inclination toward neuroblastoma formation [75].

Several studies have explored the utility of allogeneic stem cells, which may provide a solution for immediate nerve repairs with conduits [76–78]. One study seeded a conduit with embryonic stem cells (ESCs) to bridge a nerve gap spanning 10 mm. Immunostaining following regeneration revealed properly differentiated myelinating cells and a uniform connection extending from the proximal to distal stumps [76]. ESC-derived motor neurons injected into tibial nerves of mice resulted in a reduction in muscular atrophy [77]. Umbilical cord mesenchymal stromal cells (UC-MSCs) and umbilical derived cells with stem cell properties (UC-SCs) have been used in conduits to regenerate an 8 mm nerve gap in rats [79].

d. Immunomodulation.

The quality and rate of nerve regeneration is greatly improved when there is a minimal amount of scar tissue in the nerve gap. Peripheral nerve surgeons often perform neurolysis to free up nerves from scar tissue to promote regeneration [80]. External neurolysis treats scars forming on the outside of the nerve epineurium, while internal neurolysis removes intrafascicular scars. Both treatments attempt to prevent axonal constriction to allow nerve regeneration. Over-aggressive scar excision can impair the blood supply to fascicles and thus counteract its benefits. Modulating the inflammatory process to prevent scar formation for peripheral nerve repair would enhance nerve regeneration without compromising existing structures.

The key for PNS regeneration is based on myelin debris clearance. In injured CNS, myelin debris can persist from months to even years after injury and it creates a major roadblock for brain and spinal cord repair [81]. In the PNS, however, inflammatory cells such as monocytes and macrophages quickly arrive at the damaged site to begin myelin debris clearance by phagocytosis and/or autophagy which results in quicker nerve regeneration [13]. Moreover, the lesser known tissue resident glial cells, Schwann cells and perineural cells, also contribute to the myelin debris removal [13]. Lutz et al. showed that myelin clearance was dependent on TAM (Tyro3, Ax1, Mer) receptor mediated phagocytosis in a mouse model [81]. Even though past studies have uncovered several cell types that contribute to this recovery process, the recruitment of these cell types into the damaged site remained elusive.

After PNS injury, neurons quickly change their activities to promote a secretory type. Monocyte descendants, especially macrophages, quickly enter the injured site to remove inhibitory degrees and enable new exons to sprout into the degenerated nerves directed by the band of Büngner [82]. It has been known that specific macrophage types (e.g. M1, M2) can drive either the healing or inflammation response [82]. While inflammatory subtypes secrete inflammatory cytokines, M2a and M2b macrophages secrete anti-inflammatory cytokines to encourage angiogenesis and matrix forming [82].

Besides M2 macrophages, T cells and monocytes with certain receptors also play a role in anti-inflammatory PNS healing. Studies have shown that certain chemokines can induce nerve repair by increasing pro healing inflammatory cells [83]. In particular, IL-4 has been shown to have a CD4^+^ T_H_2 dependent functional tissue restoration depending on the mTOR/Rictor-dependent pathway [83]. Furthermore, monocytes with high CD43, high CX3CR1 and low CCR2 have known to induce PNS healing by enhancing debris clearing [81]. Mokrram et al. reported that fractalkine increases macrophages activity by activating CXCR1 receptor, and increases the ratio of pro-healing macrophages to total number of macrophages [82]. In fact, fractalkine treated scaffolds outperformed IL-4 treated scaffolds in PNS healing using a mouse model [82]. As such, integrating chemokines such as fractalkine or IL4 into peripheral graft have the potential to enhance PNS healing in animal models [81, 83].

Interestingly, antibody produced from B cells also plays a role in myelin debris clearance. Vargas et al. has showed that natural antibody accumulates at PNS damage sites, and that B cell- deficient mice exhibit impaired axon regeneration after sciatic nerve damage [84]. This suggests that the humoral immune system induced phagocytosis also plays a role in nerve regeneration. However, a more recent study showed that adaptive immunodeficiency Rag −/− had an increase in axonal degeneration and increased healing due to increase in macrophages to compensate for the reduced T and B cells [85]. Given the contrasting and possibly conflicting results between humoral immune system and PNS healing, more studies are needed to confirm their relationship.

One part of PNS regeneration that is overlooked is immune cell-to-cell communication. One method that immune cells communicate is by releasing exosomal microRNA (miRNA) to enhance or reduce PNS healing. For example, miR-340 from macrophages is shown to boost Schwann cell debris clearance [86]. While in a rat model, miR-223 from M2 macrophages have been shown to inhibit cell migration and proliferation by downregulating the expression level of NGF and laminin [87]. Therefore, there is a potential to use exosome to deliver miRNA to promote PNS healing.

Overall, humoral, adaptive and innate immune systems play a significant role in PNS regeneration. By manipulating different aspects of the immune systems through cytokines, chemokines or miRNA, it is possible to enhance PNS regeneration along the nerve conduit.

### Engineering approaches

- Engineered microenvironments

Engineered biomaterials used to manufacture NGCs need to promote proper nerve growth down the graft and into the distal nerve segment, while avoiding incitement of inflammatory host responses. This is accomplished through the selection of cell-interactive biomaterials as the mainstay of the grafts. Natural biomaterials found in the tissues do not elicit inflammatory responses. Structural molecules including glycoproteins, collagen, and polysaccharides help maintain tissue shape and function [88]. These biomacromolecules have shown promise in producing results similar to autologous nerve grafts in animal models, but more research is needed to determine if this will prove true in humans [69]. Biomaterials proven favorable for vascular and neural tissue regeneration with in vitro studies may surprisingly impair regeneration during in vivo studies [89], which increases the difficulty of selecting materials that translate from in vivo studies to animal models and finally human trials.

Biodegradable polymers at particular rates will be advantageous for effective nerve regeneration in native tissue. Grafts that are more porous have been shown to degrade at a slower rate than more dense grafts but with a faster surface degradation [90]. Longer degradation times may reduce the ability of the nerve to regenerate. On the other hand, grafts need to allow diffusion of oxygen, water, and nutrients to promote nerve growth [91, 92]. This requires research into the porous nature of grafts to optimize the compromise between permeability and degradation. Failure to strike this balance may interfere with the nerve regeneration process such as allowing increased infiltration of fibroblasts in more porous conduits [93].

Nerve axons also need to penetrate the substance or lengthen along microchannels within the NGCs, which means the material has to maintain its structure. It has been shown that variations in microstructure such as inner surface-area-to-volume ratio can differentially affect the growth of axons [94]. A recent study explored single lumen and multi lumen NGC designs. Single lumen has shown benefits for shorter (< 30 mm) lesions, while multi-lumen designs show no improvement in outcomes when compared with single lumen NGCs [95]. This may have occurred due to reduced permeability, flexibility, or just variations in the materials used in the actual NGCs.

Another consideration for NGC construction is manufacturing methods. Many processes exist for generating a variety of heterogenous mediums making it a significant decision in NGC design. Electrospinning, porogen leaching, and rapid prototyping are just a few of the methods used to create NGCs [38, 51]. Rapid prototyping is a less established method but has enhanced the reproducibility of computer-driven graft designs but is limited by its high costs [90]. Biomaterials used for NGCs also need to be compatible with the chosen technique. For example, certain fabrication techniques have made chitosan deleterious to nerve regeneration by inducing massive foreign body reactions after suturing to the nerve stumps [96]. While the effectiveness of the material will likely have more of a determination on what method is chosen, factors cost, availability, and large-scale production will influence feasibility for healthcare systems implementing this technology.

b. Electrical stimulation and conductive scaffolds

Electrical stimulation (ES) of transected nerves has emerged as an effective therapy to improve axonal outgrowth and reinnervation [97]. Several studies have reported accelerated axonal extension and increased twitch force following ES [98, 99]. More recent reports have provided further insights into the mechanisms underlying ES and nerve regeneration [100]. Effective ES relies on a low-frequency stimulus (20 Hz) within an hour of primary surgical repair of nerve transections [100]. A RCT using brief low-frequency ES resulted in early and complete reinnervation following carpal tunnel release surgery [101].

Tissue engineers have leveraged the effectiveness of ES to create conductive biomaterials for constructing NGCs [95]. Synthetic conductive biomaterials propagate electrical signals to stimulate regenerating axons [102]. Polypyrrole (PPy) is a conductive polymer utilized in recent bioprinted conduit designs. One major limitation to PPy is its poor biodegradability, solubility, and flexibility [103]. Yet, animal studies showed PPy sustained sciatic nerve regeneration [102–104] resulting in functional recovery approximating autologous grafts [104]. Several research groups have shown that PPy scaffolds can orient neurites and increase their number and median length [104–106]. Another promising conductive biomaterial is carbon nanotubes (CNTs) [107, 108]. CNTs or graphene-based nerve conduits integrate with native tissue and do not elicit an immune response after implantation [107]. Nanofabrication is opening up the possibility for more effective electrodes to pair with NGCs [109]. The combination of electrodes and NGCs is referred to as peripheral nerve interface devices that further enhance signal propagation for regenerating nerves [110]. Peripheral nerve devices are produced when NGCs ensheath metal electrodes comprised of nanoparticles during the 3D printing process [111, 112]. Advanced ES methods are required in conduit design to include compact electrodes that effectively integrate with more biodegradable conductive conduit materials to improve performance. Synchronizing electrical stimulation with biomaterial porosity and degradation accelerates axonal regeneration, but at the risk of exaggerated sprouting leading to entrapment. Synchronizing these conduit features remains a challenge for clinical translation.

c. Customizable and personalized NGCs

A major limitation in advanced NGC biofabrication is the ability to fabricate complex architectures, adjustable biomaterials, and customizable morphologies to accommodate different nerves and different patients [113]. Brachial plexus surgery has been overall disappointing in large part due to the inadequacy of nerve grafts to match complex anatomy and the proximity of the injury. Standard treatment calls for an ulnar nerve or sural nerve transfer for intrafascicular graft repair [114, 115]. Recently, systematic analyses propose using a dual nerve transfer for restoring shoulder abduction compared to single nerve transfer, nerve graft, or combined nerve graft and transfer [114, 115]. Developing NGCs that can adapt to the patient variations in injured nerves, local vasculature, and fascicular architecture would provide an edge to current standards of treatments for these complex injuries. Surgeons typically delay intervention by three to six months to assess for spontaneous recovery, which provides ample time for constructing customized conduits. One study used extrusion-based 3D printing to develop a personalized NGCs reflecting patient-specific macrostructural nerve morphology (shape, diameter, length) and biomimetic microchannels [67]. The downside to extrusion-based 3D printing is its low printing resolution, slower print speeds, and artifacts [116, 117]. Addressing these limitations is necessary to scale biomanufacturing operations to optimize NGC performance and meet the volume of health systems for acute and chronic nerve repairs. Researchers can combine the imaging information of computer tomography (CT), magnetic resonance imaging (MRI), and computer-aided design (CAD) for macrostructural dimensions and geometries, while 3D reconstructions of nerve histology slices provide the microstructural pattern. Current treatment guidelines utilize magnetic resonance neurography (MRN) to locate nerve injuries and characterize the extent of injury. Tissue engineers can process these scans for preparing a blueprint to fabricate NGCs to determine nerve diameter, gap length, and nerve shape. Generating patient-specific fascicular architecture for NGCs may require sampling complete nerve injuries during exploration if primary repair without tension is not possible [118, 119]. This may be feasible in the circumstances of certain nerve transections or following nerve tumor resections. A more promising solution would be the arrival of high-powered (7 T) MRI scans that can visualize fascicular patterns within nerves [120]. Fascicle matching will require coordinating clinical management and advanced bioimaging and tissue processing tools to generate microchannel facsimiles of patient tissue [116, 118].

Matching implant and host tissue properties is important for NGCs to reduce the risk of compressive or tearing of regenerating nerves [119–121]. Zhu et al. proposed a digital light processing (DLP)-based rapid continuous 3D-printing method to fabricate customized NGCs with material properties corresponding to the injured tissue [113]. 3D bio-printer specifications will need to precisely control mechanical properties of 3D prints to match the varying viscoelasticity of peripheral nerve tissue [122–124]. Another study incorporated multiple design elements (geometry, NTFs, anisotropic gradients) effective in nerve regeneration within a single conduit [125]. Personalized conduits will need to build on these recent advancements by incorporating conductive materials, programmed stem cells, nerve differentiation, and vascularization into a single design. Combining these features will serve to increase nerve gap regeneration size and improve functional recovery.

#### Surgical intervention

- Vascularization

Peripheral nerves receive their blood supply from small vessels entering the epineurium (intrinsic) from neighboring feeding arteries (extrinsic). The intrinsic blood supply is important for regeneration because it allows large molecules, growth factors, immune cells to enter the endoneurial space after the blood-nerve barrier breaks during Wallerian degeneration [126]. Vascularity of nerve grafts has become a greater focus of nerve graft research [62]. The importance of creating grafts with vascular networks is the need to increase the effectiveness of longer graft segments. It is thought that one of the limiting factors is the ability to supply enough nutrients and oxygen to facilitate axon growth. Research has shown that vascular networks within NGCs can be enhanced in the early post-transplantation period by including vascular bundles in the graphs [127]. Consequently, increased vascularity of nerve grafts has been demonstrated to improve overall nerve regeneration and axonal elongation [128, 129]. They have also been shown to significantly increase nerve diameter, neuron number and electrical conduction in comparison with non-vascular silicon grafts [62]. Vasculature networks enhance nerve repair by transporting oxygen and nutrients, sustaining axon survival, and directing axonal extension [62, 130]. Activating these cellular processes is crucial in long-standing nerve injuries where reduced perfusion creates resistant microenvironments for axiogenesis [131]. Surgeons anastomose nerve graft vessels to recipient vessels to maintain perfusion and prevent necrosis during nerve repair [132]. Vascularization is an attractive graft feature for chronic injuries or delayed surgical repairs characterized by low vascularity because these grafts could accelerate the rate of axonal elongation [133, 134]. Synthetic nerve graft performance has also been found to improve when paired with local vasculature [135–137]. However, studies suggest that while these vascularized grafts can improve reinnervation over non-vascularized grafts, they have yet to equal non-vascularized graft performance [135]. A major objective in surgical nerve repair is avoiding damage to extrinsic vascular supply feeding intrinsic vascular networks and collateral circulation because it can result in ischemia, nerve death, and scar formation [138]. Providing surgeons with a vascularized graft would allow them to perfuse the injured nerve and improve the success rates of nerve conduits. In the future, the combination of vascularized nerve scaffolds can provide the foundation for complex nerve tissues e.g. skin grafts or neuromuscular grafts.

b. Nerve differentiation

One of the main shortcomings of nerve regeneration following PNI is the inability to recover normal motor and sensory components [51]. Mixed nerve injury repairs utilize nerve transfers from sensory nerves such as the sural nerve. For example, nerve grafts using the sensory sural nerve display inferior regeneration when used for mixed or motor nerves [139]. Nerve regeneration without precision in motor and sensory differentiation, guidance, or target reinnervation is a major difficulty for current treatment options [51]. Brushart observed that mixed nerves display preferential motor reinnervation (PMR) where regenerating motoneurons reaches their appropriate motor pathway and target when compared to sensory pathways [140]. In other words, when motoneurons are provided equal access to motor and sensory pathways motoneurons favor the motor pathways. The components generating this biological preference include pathway-specific support cell phenotypes [72], NTF composition [141], and both pathway-specific ECM proteins and architecture for sensory and motor trajectories [139].

The concept of modality-specific Schwann cell phenotypes originated from PMR research, which found that distinct motor and sensory Schwann cells existed for individual pathways [141]. Researchers have found differential NTF expression for motor and sensory Schwann cells [72, 142]. Microarrays has revealed that pleiotrophin (PTN) is specific for Schwann cells in motor pathways, which has neurotropic and neurotrophic effects [143]. PTN or heparin binding neurotrophic factor expressed in Schwann cells, endothelial cells, and macrophages promotes elongation of motoneurons [144]. Conversely, the same studies found sensory axons grew away from PTN gradients. Another study found sensory neurites have the propensity to grow towards elevated levels of BDNF and NGF [145]. Misdirection of motor and sensory pathways is a persistent problem for nerve regeneration and repairing mixed nerve injuries [141]. Besides growth factor composition and Schwann cell phenotype, Madison et al. found the most important determinants for peripheral nerve regeneration was muscle contraction in the distal stump along with Schwann cell density in the distal stump [146]. The development of NGCs with the capacity for sensory, motor, or mixed regeneration would provide a crucial advantage over current treatment options for mixed nerve injuries [67].

### Challenges to incorporate multiple biological and engineering factors for surgical intervention

The first section identified the key components and strategies used to regenerate peripheral nerves. Tissue engineering deploys the biotechnology toolkit to embed biological components into engineered structures. Harmonizing biological components (genotypes, stem cells, growth factors, immunomodulation, vascularity, etc.) and strategies (gene-editing, cell culturing, cell-signaling, biomaterials, bioprinting) with native conditions (injury type, nerve type, nerve dimensions, gap length, anatomy, and host-tissue receptivity) is a major challenge for systematizing the biofabrication process. Several constraints impede progress in this area, namely, current characterizations and approaches to regeneration, cost of entry into biofabrication and biotechnology, and biomedical regulations.

Current surgical management for PNIs has several drawbacks. Nerve grafts such as allografts and xenografts require immunosuppression and expose the patient to risks of cross contamination, immune rejection, and infections passed from the donor [147–149]. Immunosuppression also makes the patient more susceptible to infections postoperatively. AvanceⓇ is one such FDA approved allograft that has been decellularized and processed for implantation. AvanceⓇ has been shown to be effective for nerve repairs spanning 1-2 cm, and actually outperformed available second-generation conduits [150]. However, Avance does not yield the regenerative effects produced by isografts. Autografts require sacrificing native nervous tissue that usually result in sensory deficits and are often insufficient for reasons pertaining to nerve size, length, nerve complexity (plexuses), nerve type (motor, mixed), etc. [151]. First- and second-generation NGCs have been developed and approved by the FDA for nerve repair [152]. First-generation NGCs are generally preferred for long gap repairs exceeding 4 cm [152]. Their lumens lack supportive elements (e.g. cells, proteins, growth factors, blood products) that newer synthetic conduits incorporate. One such example is the SaluTunnel™ made of nonresorbable PVA hydrogel. SaluTunnel is not validated by clinical studies making their clinical use uncommon [153]. Second-generation nerve conduits are made from resorbable material with greater biocompatibility (e.g. type I collagen, poly-DL-lactive-co-caprolactone (PLCL), polyglycolic acid (PGA)). Similar to first-generation nerve conduits, second-generation sconduits have empty lumens without supportive elements. Neuragen™ Nerve Guide is an FDA approved resorbable implant derived from bovine tissue. It is a porous Type 1 collagen tube designed to provide a protective environment for nerve repair. Neuragen provides an adequate solution for repair in nerve gap lengths between 1 and 2 cm based on several studies [154, 155]. Overall, deficiencies in nerve grafts and first- and second-generation NGC design make tissue engineered NGCs more attractive for treatment of long gap PNIs and enhancing short gap repairs.

Tissue engineering and biomanufacturing is in its infancy and presently lacks standardized facilities and operations for scaling within the medical industry. Internal constraints stem from a deficiency of systems in tissue engineering leading to disjointed research disciplines (genetics, cell biology, bioengineering, medical experts). Current research largely identifies components of biological structures and attempts to include them into designs intended regenerate tissue. This approach ignores the scalar hierarchy of biological form and function where organization and timing of component interactions at each level (genes, proteins, cells, tissue) is just as important as the components themselves for higher-order functions to emerge. To combat the complexity and imprecision of biofabrication, we propose machine intelligence be employed at each stage of the NGC biomanufacturing process. The following section provides examples of machine intelligence augmenting tissue engineering from literature with an emphasis on peripheral nerve regeneration. These areas address internal constraints (biomaterial design, stem cell editing, graft performance, bioprinting) and external constraints such as the financial barriers (equipment, personnel, facilities) and regulations to biomanufacturing. In an effort to integrate tissue engineering with machine intelligence, we reviewed the status quo of ML and neural networks for NGC design and PNS regeneration in the following sections.

### Machine intelligence approaches to tissue engineering

- *Machine intelligence and adaptation to nerve regeneration*

With the advancement of computer hardware and the emerging of technologies in deep learning, Artificial Intelligence (AI) has become one of the fastest growing technical fields and has been adopted across many application domains including healthcare. AI based technologies have the potential to transform the healthcare and the number of AI based solutions for healthcare approved by U.S. Food and Drug Admission had increased to 26 by July 2019 [156].

Deep learning as a subset of machine learning (ML) which is in turn a subset of Artificial Intelligence (AI), has become so popular and led to the AI explosion in the last five years [157–159]. Artificial intelligence (AI) can be defined as the science and engineering of making computers behave in a manner like humans. AI is a multidisciplinary topic in data science, which merges computer science, cognitive science, and mathematics to develop processes, algorithms, and systems to achieve human intelligence by machines. Another topic closely associated with AI is ML, it is an approach to achieve AI by parsing data, learning from that data and then applying what they have learned to make an informed decision. With ML, machines are trained using large amount of data and algorithms such as Artificial Neural Networks (ANN) to extract and represent knowledge from the data. ANN has been around since early 1990s, but in a limited fashion (e.g. three layers—input, hidden, output) due to the limitation of computing power. With the emerging of powerful computer chips, microprocessors and graphics processing units (GPU), Convolutional Neural Networks (CNN) have emerged. CNNs can be stacked over 100 layers deep. This is the origin of Deep Learning. It is a more advanced technique for ML implementation. With a multitude of layers, the deep neural networks can be used to tackle complex data and enable many applications of DL in various domains.

Deep Learning has now become the main driver for many new applications. It can be used for object detection, classification and regression. Some state-of-the-art deep learning models for the object detection include: R-CNN [160], Fast R-CNN [161, 162], Faster R-CNN [162] proposed by Ross Girshick et al. at UC Berkeley, and Yolo [163] presented by Joseph Redmon et al. from Facebook AI Research; the state-of-the-art deep learning models for the classification include AlexNet designed by Alex Krizhevsky et al., [164] and VGGNet invented by Visual Geometry Group from University of Oxford [165], ResNet proposed by Kaiming He et al. at Microsoft Research [166], and DenseNet designed by Gao Huang et al. [167]. For the regression, the final layer of the above models for the classification can be replaced with a fully connected regression layer with linear or sigmoid activations. Some traditional regression methods may also be useful for solving problems in NGC design, such as ordinary least square and gradient descent based linear regression, and Gauss-Newton, Gradient descent and Levenberg-Marquardt algorithms based nonlinear regression. Recent work on Bayesian neural networks shows the feasibility of estimating uncertainty due to the lack of training data. This is important for medical applications because of the safety requirements. Research demonstrates that some new network architectures generalize well even with less training data, such as capsule networks [168].

To achieve the promises of peripheral nerve repair using NGC and digitally translate a blueprint of design to regeneration [169, 170], it will require harnessing big data quantitatively and predictively in the lab and clinic for every aspect of the regeneration. These include but are not limited to AI-based engineering design tools for NGC. The emergence of deep learning technology can lend itself to many aspects in biofabrication including regulatory compliance, stem cell priming and selection, growth factor concentrations, favorable biomaterial properties, regenerating multiple tissues in a single scaffold, decoupling multiple characteristics for a regenerative outcome, selecting patient-specific NGC features based on parenchymal composition, nerve types, modes of injury, genetic variations, enhance device testing, and improving AM operations. We will explore where current research has merged machine intelligence and nerve regeneration and where machine intelligence and tissue engineering can interface to accelerate innovation.

To demonstrate how to use ML for the regeneration, we selected a few cases which provide examples of using ML for improving conduit performance via modification of biomaterial properties, growth factor considerations, or improving stem cell cultures through gene editing. We will start with an example illustrating how to use our proposed ML method for personalized, AM of nerve guidance conduit.

b. *ML for optimal NGC design for AM*

Poly (glyerol sebacate) (PGS) is an emerging promising flexible biomaterial for nerve repair. The photocurable of PGS can be used for light-based AM of NGCs [171, 172]. Chemical and mechanical characterization results showed that PGS became stiffer with increasing degrees of methacrylation, and its surface became more hydrophobic and the degradation rate decreased. The mechanical properties in the range of soft tissues, and the degradation from weeks to months can be tuned to accommodate both fast and slow regenerating tissues. Depending on the length of the nerve gap of individual patients, the material can be tuned to degrade at an acceptable rate which is long enough for regeneration and maturation, yet quick enough to minimize long-term inflammation. Conduit size is also an important factor for regeneration and balancing the internal diameter to allow the injured nerve to expand without compression while preventing ingress of surrounding tissues.

For the optimal design and 3DBP, we need to predict the percentage methacrylation ***P*** required for the 3DBP of an NGC for a particular patient based on the nerve gap of the patient *g*, the time required for the nerve regeneration and maturation *t* with concurrent degradation of the NGC, the diameter of the conduit *d*, and the thickness of the conduit *w*, as shown in the function below.

$$ \boldsymbol{P}=f\left(g,t,d,w\right) $$

Because the relationship among the percentage methacrylation and other factors expressed in the above function can be highly non-linear, an empirical or phenomenological constitutive equation is most often used to model such a relationship and is fit using multivariate non-linear regression. However, the regression is usually restricted to certain conditions. To make the model generalize well and adapt properly to new, previously unseen data, ML can be used to model the highly non-linear relationship and predict the percentage methacrylation using the following parameters as input: the nerve gap of individual patients, the time required for the nerve regeneration and the conduit dimension. The predicted percentage methacrylation can then be used for an optimal design of the NGC. For example, when using TensorFlow platform to build a model to predict the percentage methacrylation, we will need to provide the model with a dataset including sample data record in the following format: gap, time, diameter, thickness, percentage methacrylation. The dataset can be acquired from experiments. The more training samples, the better the performance of the model is. However, in data-limited situations, generative adversarial networks (GANs) can be used to generate new sample data which are realistic enough and proved to be really useful. The dataset can split into a training and a test set. We can use the training set to train the model and the test set for the final evaluation of the model. The value range of the gap, time, diameter and thickness can be different, so normalization of these inputs can be conducted to make the training easier. A sequential model can be used with two densely connected hidden layers and an output layer to predict a single value for percentage methacrylation. The model can then be trained using the training set and evaluated with the test set.

c. *ML to optimize cell phenotype*

Besides using ML to enhance NGC designs, it can be used to optimize the biological components comprising NGCs. Gene editing tools such as CRISPR-Cas9 work by attaching protein Cas9 to a specific genetic target using RNA guides. Effective gene editing requires selecting RNA guides that are precise to the particular gene region of interest (on-target effects), while reducing the RNA guide’s compatibility for other segments within the genome (off-target effects). Machine Learning programs have been developed to improve both on-target efficiency (e.g. Azimuth) along and off-target prevention (e.g. Elevation) using a regression tree with boosting model [173]. Predictable and precise gene editing tools can lend themselves to modifying cells to be more effective for nerve regeneration. This can occur by targeting genes to increase the expression of Schwann cells, programming nerves to differentiate as motor or sensory nerves, enhancing immunomodulation of support cells, or regulating the time of regenerative events. Another research group, developed a novel method for making template-free genome editing more precise using a ML algorithm (e.g. inDelphi) to predict and design RNA guides with preferred repair genotypes [174]. Training data was acquired and the algorithm was refined to separate repair outcomes into deletions and insertions based on their projected repair mechanisms [175]. The mature program was able to correct over 183 pathogenic human alleles in greater than half of the edited products, designed predictable 1-base pair insertions, and enhanced success rates. Besides the use of ML for genetic editing, ML can also make predictions about cell phenotypes and gene-regulatory networks to further improve cell selections incorporated into the end product [176]. Recent developments in stem cell technology have begun to merge big data in the form of genomics, transcriptomics, proteomics, epigenomics, and metabolomics with ML to safely produce cells that best match the target patient and the regenerative process of interest [177]. Standardized performance of stem cells will improve the effectiveness of 3D bioprinted products and their reproducibility.

d. *ML for quantitative assessment of neuronal response to NGC material properties and architecture*

Quantitative assessment of neuronal response to the surface topology is important [178, 179]. Advanced imaging and cellular image analysis can be used to objectively quantify both neuron and neurite outgrowth using a number of measurements of neurons and neurites [172, 179, 180]. The measurements include number of neurons, total and average neurite outgrowth, total and average neurite area, total and average number of segments, average branching layers, the longest neurite from a neuron, total and average number of roots, total and average number of extreme neurites, total and average number of branch points etc. The cellular image analysis can also help quantify the angle of neurite outgrowth, [181] number of axons, axonal growth and the alignment of neurite [182]. Based on the measurements on neuronal response, ML can help with the optimization of surface characteristics. For examples, studies show that topographical grooves with different height and inter-groove spacing have different impacts on neurite outgrowth. ML can help optimize these parameters for guiding neurite outgrowth [181]. Similar to the example for the prediction of the percentage methacrylation, a ML model can be built to model the relationship between the neurite outgrowth, and the surface topographies of NGC.

Nascent neurons elongate and elaborate fine and fragile cellular extensions that form circuits enabling communications and find paths to distant targets. As a result of post-developmental neuronal damage, the cues for reinnervation are no longer active. Advances in biomaterials are enabling fabrication of microenvironments that encourage neuronal regrowth and restoration of function by recreating these developmental cues. The combination of topographical and electrical cues greatly improves length of neurite extension. Electrical stimulation can be further integrated into the scaffold by choosing a conductive base material. However, it is quite challenging to manipulate and integrate these elements in different combinations to generate new technologies to enhance neural repair. An ideal substrate for effective repair should take into account the combination of topographical, chemical, electrical, and mechanical properties of the substrate [183, 184]. Similar to the example for the percentage methacrylation prediction, ML can be used to optimize the engineering of the substrates by identifying the optimal combination of topographical and electrical cues.

e. *Application to AM and 3DBP*

Manufacturing three-dimensional (3D) structures in nerve regeneration is a growing activity amongst tissue engineers. Machine intelligence is beginning to standardize AM methods in metal printing and more rarely bioprinting. Bock et al. break down these approaches into descriptive, predictive, prescriptive categories for microstructure, process parameters, mechanical properties, and performance in metal printing [185]. Descriptive approaches explain how structural patterns, material properties and printing processes relate to material performance. Descriptive tasks emphasize pattern recognition and correlation to establish data-driven workflows in AM, which is currently lacking in bioprinting [186]. Materials have microstructural properties that can be quantified in terms of size, shape distribution, anisotropies, and component geometries then analyzed using principal component analysis, factor analysis, and independent component analysis to discover techniques that reduce dimensionality to extract process-structure linkages [186]. Thus, descriptive approaches identify and validate input (processes) leading to specific outputs (microstructural patterns). Descriptive characterizations of microstructures remain a significant challenge in materials science. Material informatics are beginning to develop objective measures to distinguish between heterogenous materials [186]. For instance, Altchuch et al. mined materials for structural features based on pore size, pore shape, degree of porosity to estimate the features of real membranes. Finally, descriptive methods can be used to characterize the effect of stress/strain (macroscale conditions) on bone to compute mechanical property changes on trabecular bone at the mesoscale using finite element stimulation and ANN [187, 188]. These techniques could lend itself to electroconductive materials to improve its biodegradability and elasticity.

Additionally, predictive approaches forecast the performance of 3DBP products based on minute changes made to microstructures, properties, or printing process. Predictive approaches inform the engineer what parameter is most likely to produce a desired outcome. This technique focuses and limits our tests to those with the greatest likelihood of being significant, while minimizing utilization of costly resources. Predictive tasks employ ANNs to predict material properties (width, height, strength, degradation rate, gf concentration, cell deposition) from specific process parameters (print speed, extrusion rate, temperature profile, nozzle-to-plate distance, resistance to flow). Xiong et al. implemented a similar ANN scheme for metal AM by relating process parameters (welding speed, wire feed rate, arc voltage) to height and weight of deposited layers [189]. Another interesting study used a CNN architecture relating microstructural (lower-level) material information to macroscale (higher-level) properties [190, 191]. This case study found a 38% increase in predicting macroscale strain component from microscale volume elements compared to previous models [191]. When experimental data is lacking, generative ML models can be used to create authentic material samples to training purposes [192].

Lastly, prescriptive approaches provide input recommendations to produce certain outcomes. For example, GF concentration should exceed this specified amount (in ng or μg) to regenerate nerve across distance (in mm). In this case, machine intelligence identifies which process parameters should be applied to acquire a specific material property. Chupakhin et al. developed an ANN to optimize residual stresses profiles in materials after laser shock peening [193]. These strategies could be enlisted to propose specific processes and parameters that attenuate the tension between material porosity and biomaterial degradation and enhance nerve regeneration. Prescriptive techniques could advise engineers the best material properties for specific regenerative applications by generating and refining random microstructure-property pairs to select optimal results [191]. Liu et al. took a ML-based approach to refine search paths and decrease search regions when designing alloys [191]. This approach lowered costs for finding optimal solutions. Similarly, a prescriptive framework used phase field simulations to reduce the number of experiments needed to establish process-structure-property linkages [190].

Machine intelligence can also be used to improve bioprinter performance. Researchers have used ANN to investigate orientation, the delay between layers, and layer thickness as inputs in relation to porous structures and scaffold strength as outputs [194]. The algorithm determined the optimal 3D fabrication parameter settings for creating optimal porous structures for bone tissue regeneration. Gu et al. performed finite element analysis using a ML algorithm to identify superior biomimetic microstructures focusing on material and geometric properties [195, 196]. ML-based design created diverse microstructural patterns and materials that were proved effective after 3D printing and testing. This research could extend to nerve tissue engineering by optimizing microchannel dimensions, arrangement, and conduit physical properties for regenerating sensory, motor, or mixed nerves. Another ML algorithm called Non-dominated Sorting teaching-learning-based optimization (NSTLBO) proposed solutions to multi-objective optimization problems in rapid prototyping processes to minimize cost of production with product quality, energy consumption, and mechanical properties [197]. Similarly, other research groups have augmented bioprinters using ML, specifically to optimize their process parameters and improve printer consistency [198–200]. Drop on demand bioprinting must balance drop speed, drop volume, and satellite generation [201]. Machine learning using the Adam algorithm was found to improve printing precision and stability after training [201]. Tolerancing will become a major issue for 3D bio-printed NGCs because they contain multiple materials, biological components, and geometric variation. Tolerancing refers to the control or limiting of geometric variation of the final product. ML has helped limit variation for prints in AM and provides another role in biofabrication standardization [113]. Computer vision has been utilized in AM processes to detect defects during the build process [202]. After proper training, the classifier was able to detect defects with 80% accuracy. Machine sensors can analyze acoustic signals or video recordings to assess the quality of printed layers by identifying aberrant features [203, 204]. Machine vision can also fine-tune the electrohydrodynamic printing (EHDP) by monitoring cone shapes for more precise scaffold biofabrication [205]. Digital microscopic imaging combined with CNN Developing automated inspection systems using machine intelligence can reduce print variation experienced during conduit production. Advancing ML in AM processes is critical for scaling production, ensuring quality control, and minimizing cost-of-production in healthcare settings [203].

f. *Enhancing graft performance by neural networks*

Matching a particular nerve graft to a patient is an important function that machine intelligence can perform for PNI [206]. Many injuries may not require all of the features mentioned in the previous sections, making graft fabrication faster and cost-effective by tailoring features for the particular patient and injury. Conforth et al. developed a Swarm Intelligence based reinforcement learning (SWIRL) to predict graft success under specific conditions. SWIRL is an ANN that was trained to identify the best combinations for a given outcome [206]. Thirty variables were classified based on biomaterials, ECM proteins, GFs, cells, number of lumens, surface quality, and filling types. Outputs considered were regeneration length, ratio of actual length to critical length, and graft success estimation. SWIRL was able to obtain a greater than 92% accuracy for graft selection.

Koch et al. used a calculated ratio of gap length divided by the graft’s critical axon elongation as a measure of nerve regenerative performance [207]. Bootstrap aggregated neural networks were used to predict NGC performance using 40 features as inputs. The selected features included parameters known to impact the performance of the conduits e.g. material process parameters—phase separation and hydrogels, structural parameters—internal diameter, conduit length, wall thickness, and selected growth factors—NGF, NT3, BDNF [207]. Experiments show that the bootstrap aggregated neural networks outperform the SWIRL in terms of prediction accuracy [207, 208]. The improved accuracy can help engineers make more impactful design decisions to optimize NGC performance.

g. *Application of ML to regulatory compliance*

FDA-approved devices for treating PNIs are typically derived from natural biomaterials (collagen), synthetic materials, or allogeneic tissue [62]. These devices fail to repair nerve gaps exceeding 3 cm. This makes the development of alternative devices desirable with customized material compositions using bioactive components [209, 210]. Advanced therapy medicinal products contain a difficult burden of regulations to translate nerve conduits into clinical practice. Toxicity and biocompatibility tests along with phase I and II trials are costly to perform. Current FDA device regulations will require oversight across multiple stages during manufacturing including stem cell processing, device storage, and pharmaceuticals. Product developers must consider product shelf-life of 3D bio-printed conduits for commercialization. Standardized manufacturing guidelines do not exist for NGC production. In general, there is a requisite for FDA-approval to implement 3D bio-printed devices on a clinical scale [211, 212]. Machine intelligence can provide several useful tools to maintain compliance with regulations to increase the likelihood of devices reaching the testing stage. ML applications can be developed to identify requirements and promote compliance with regulations [213, 214]. One algorithm works by matching regulatory requirements with engineering processes to simplify product development, identify deviations from regulatory expectations, and minimize administrative and fabrication costs [214]. Nomos3 is a modelling language specializing in modelling laws and regulations and performs automated compliance analysis [214]. Another group tested three different languages, namely, compliance request language (CRL), computational tree logic (CTL), and linear temporal logic (LTL) to maintain compliance with business processes, operations, and practices [215]. Compliance management frameworks inform businesses when violations to rules have occurred and what solutions can resolve violations [215, 216].

## Conclusions and outlook

**Fig. 2:**
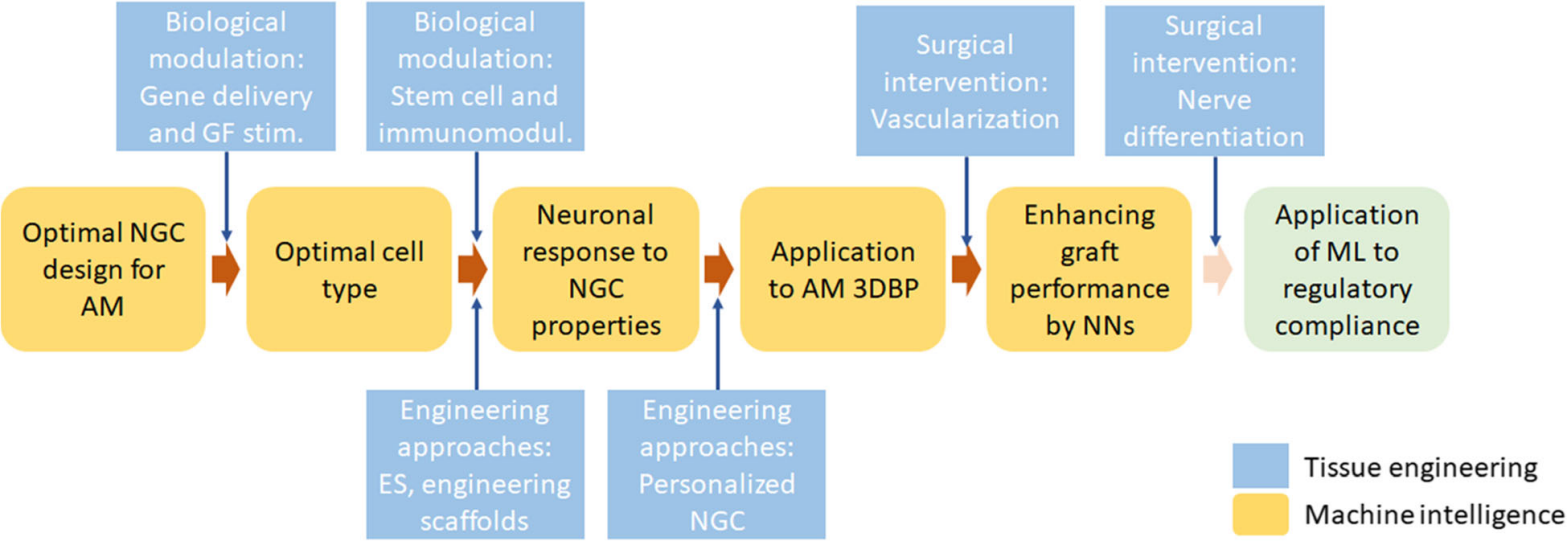
Hypothetical steps to create NGCs with the proposed integrated tissue engineering and machine intelligence approaches. The optimal performance of NGC requires a tight integration and synergy from basic science, advanced tissue engineering approaches and clinical practices with elaborated model of machine intelligence. Ample data will facilitate training and standardize the production and feedback look to fulfil the requirement of regulatory compliance

Peripheral nerve damage continues to have high incidence in the United States, as well as poor prognosis and lifelong disabilities. Due to the variety of peripheral nerve damage etiologies, treating such condition by surgical means remains a challenge. NGC provides an alternate method to tradition surgical approaches such as allogeneic nerve transfer, or in more extreme interventions, free functional muscle transfer [217]. In this article we have reviewed three categories of NGC design components: biological modulation, engineering approaches and surgical modulation. Furthermore, we examined how machine intelligence can combine all three components in NGC design and manufacturing with ML approaches. As depicted in Fig. 2, approaches discussed in each section can be integrated to produce an NGC with an optimal performance not only by design and engineering perspectives, but by clinically applicable outcomes to treat PNI. By doing so, we hope that soon personalized NGC can be manufactured efficiently and effectively with economy of scale.

As technology advances, personalized medicine increases in demand and popularity [217]. Since each patient is different in metabolism and genetics, their treatment response to biomaterials can differ significantly. Effective NGC design requires the complex understanding of cellular machinery, tissue engineering and surgical procedures, which requires a combined team of scientists, engineers and physicians. Thus, such designs incur high human cost and resources. It is more efficient to use AI to generate quick, accurate and non-bias design decisions, which can also constantly be trained and developed by retraining the AI models on new data. As such, ML can advance the regeneration of PNS in 3D biofabrication, graft performance and prediction of NGC performance.

To achieve a quantum leap toward functional tissue regeneration, we may have to look at the regeneration problems at a different viewpoint. Although the field of AI research was initiated at the Dartmouth Conference in 1956, the power of AI was grossly underestimated, followed by the AI winter. However, investment and interest in AI has taken off since early 2010 when ML showed meaningful progress to provide solutions in academia and industry with significant advancement in computing and storage hardware and the presence of big data. The machine intelligence is being adopted to almost all areas. For example, biomedical imaging [218] and electronic health record [219] are already exploiting ML and AI. Now we want to see the application of machine intelligence to a field which is somewhat less systematically explored, or traditionally more challenging, and is only dealt with mathematical and statistical modeling.

One issue to consider with AI and ML is that they require huge amount of training data sets to become viable and reliable. The emergence of big data analytics allows extraction of relevant information from different research, data base and clinical data necessary for ML. However, the ML models have been largely attributed to the quality and diversity of training data. If there is lack of such training data, data augmentation can be used to significantly increase the diversity of data available for training models without actually collecting new data. GAN can be used to generate synthetic data for training ML models. However, deep learning in biomedicine and 3D bioprinting is still in its infancy, and that there is much to understand of peripheral nerve regeneration biology. Furthermore, ML designs must also be validated in an in vivo model. As mentioned in the article, multiple groups have attempted to utilize ML for optimization of NGC designs, yet little have been done to test such designs in an animal model. While ML models can be used to predict the probability that such design will optimize in peripheral nerve regeneration, clinical trials in humans remains the gold standard for application. There must be more in vivo studies using such designs in mouse and monkeys as proof of concept, so that ML generated NGCs can lead to clinical trials.

We live in an exciting time where biological and technological advancements emerge rapidly. For example, CRISPR-Cas9 gene editing technology was discovered in 2012, while GANS was invented in 2014 [218]. Given so many significant discoveries happen in such a short amount of time, it is not hard to imagine that AI and ML can also lead to great improvements in NGC designs soon. In the future, there will be a much heavier computational emphasis in drug and treatment development [220], and that there will be horizontal integration between basic science, data science, engineering and clinical science. As deep learning develops it will transform human wellness and healthcare.

**Table:** 

Machine Intelligence for NGC Production		
Biomanufacturing Phase	Main characteristics	Refs
Biomaterial Development	GANs for data-limited situations to optimize methacrylation Monte Carlo approach to predict microstructural outcomes from AM process parameters Predicting biomaterial macroscale properties from microstructural elements	[171, 172, 186, 188, 191, 192]
Stem Cell Editing	Improving gene on-target efficiency Minimizing off-target effects Predicting phenotypic outcomes from genetic regulatory networks	[173, 175, 176]
Cell-Material Interactions	Quantitative Assessment of neurite response to biomaterial surface topology Automated assessment of neurite outgrowth and orientation	[179–181]
Digital Design	ML optimization of scaffold geometric properties	[194–196]
3DBP Process Parameters	Optimize micro-droplet generator, electrohydrodynamic, drop-on-demand, and spheroid-based bioprinters	[199–201, 205]
NGC Performance	SWIRL for predicting graft success Bootstrap aggregated neural networks for predicting conduit performance	[206–208]

## Data Availability

Not applicable.

## Abbreviations

AAV: Adeno-associated viral
ADSC: Adipose-derived stem cell
AI: Artificial intelligence
AMSTAR: A Measurement Tool to Assess Systematic Reviews
ANN: Artificial Neural Networks
BDNF: Brain derived neurotrophic factor
BMSC: Bone marrow-derived stem cell
CAD: Computer-aided design
CNN: Convolutional Neural Networks
CNT: Carbon nanotube
CNTF: Ciliary neurotrophic factor
CRL: Compliance request language
CT: Computed tomography
CTL: Computational tree logic
DLP: Digital light processing
EBM: Evidence-based medicine
ECM: Extracellular matrix
EHDP: Electrohydrodynamic printing
ES: Electrical stimulation
ESC: Embryonic stem cell
FGF: Fibroblast growth factor
GAN: Generative adversarial network
GDNF: Glial cell derived neurotrophic factor
GF: Growth factor
GPU: Graphics processing units
IL6: Interleukin-6
LTL: Linear temporal logic
miRNA: MicroRNA
ML: Machine learning
MRI: Magnetic resonance imaging
MRN: Magnetic resonance neurography
NGF: Nerve growth factor
NSC: Nerve stem cell
NSTLBO: Non-dominated Sorting teaching-learning-based optimization
NT3: Neurotrophin-3
NTF: Neurotrophic factors
PGS: Poly (glyerol sebacate)
PMR: Preferential motor reinnervation
PNI: Peripheral nerve injuries
PPy: Polypyrrole
PSC: Pluripotent stem cell
PTN: Pleiotrophin
RCT: Randomized control trials
SWIRL: Swarm Intelligence based reinforcement learning
UC-MSC: Umbilical cord mesenchymal stromal cells
UC-SC: Umbilical derived cells with stem cell properties
